# Critical assessment of day time traffic noise level at curbside open-air microenvironment of Kolkata City, India

**DOI:** 10.1186/s40201-015-0219-6

**Published:** 2015-09-26

**Authors:** Anirban Kundu Chowdhury, Anupam Debsarkar, Shibnath Chakrabarty

**Affiliations:** Department of Civil Engineering, Jadavpur University, Kolkata, 700032 India

**Keywords:** Traffic noise level, Noise climate, Traffic noise index, Correlation analysis, Principal component analysis, Background noise component, Peak noise component

## Abstract

**Background:**

The objective of the research work is to assess day time traffic noise level at curbside open-air microenvironment of Kolkata city, India under heterogeneous environmental conditions.

**Results:**

Prevailing traffic noise level in terms of A-weighted equivalent noise level (L_eq_) at the microenvironment was in excess of 12.6 ± 2.1 dB(A) from the day time standard of 65 dB(A) for commercial area recommended by the Central Pollution Control Board (CPCB) of India. Noise Climate and Traffic Noise Index of the microenvironment were accounted for 13 ± 1.8 dB(A) and 88.8 ± 6.1 dB(A) respectively. A correlation analysis explored that prevailing traffic noise level of the microenvironment had weak negative (−0.21; *p* < 0.01) and very weak positive (0.19; *p* < 0.01) correlation with air temperature and relative humidity. A Varimax rotated principal component analysis explored that motorized traffic volume had moderate positive loading with background noise component (L_90_, L_95_, L_99_) and prevailing traffic noise level had very strong positive loading with peak noise component (L_1_, L_5_, L_10_). Background and peak noise component cumulatively explained 80.98 % of variance in the data set.

**Conclusions:**

Traffic noise level at curbside open-air microenvironment of Kolkata City was higher than the standard recommended by CPCB of India. It was highly annoying also. Air temperature and relative humidity had little influence and the peak noise component had the most significant influence on the prevailing traffic noise level at curbside open-air microenvironment. Therefore, traffic noise level at the microenvironment of the city can be reduced with careful honking and driving.

## Background

Traffic noise contributes more than 55 % of total environmental noise in urban area [1–3]. It is also accounted for over one million healthy years of life lost annually to ill health and may lead to a disease burden that is second only in magnitude to that from air pollution. Long-term exposure to traffic noise is found to be associated with cardiovascular disease, cognitive impairment, sleep disturbance, tinnitus, annoyance, increased risks of all-cause mortality, mental health impairment, central obesity and non-Hodgkin lymphoma in general population [4–8]. Day time traffic noise level of more than 50 dB(A), the guideline recommended by World Health Organization for day time for outdoor living area were reported in cities like Sanandaj, Bangkok and New York [9–12]. Most of the Indian cities and towns like Visakhapatnam, Kolhapur, Asansole and Balasore have also been facing serious traffic noise pollution in last few decades due to substantial growth of new vehicles, low turnover of old vehicles, inadequate road network and urbanization [13–16]. Assessment of traffic noise level is difficult in Indian cities due to the heterogeneity in traffic and environmental conditions e.g., mixed vehicle types, congestion, road conditions, frequent honking and lack of traffic sense [17, 18]. Therefore, it is important to consider such diverse factors in monitoring and assessment of traffic noise level in the Indian context. The objective of the present research work is to assess day time traffic noise level at curbside open-air microenvironment of Kolkata city, India under heterogeneous environmental conditions.

## Methods

### The study area

Kolkata is the capital of the state West-Bengal, India and is also one of the most populous cities of the country. The city is bounded to west and north-west by the river Hoogly. The city has a tropical savannah climate with a marked monsoon season. The city is divided into five major geographical regions namely, east, west, north, south and central Kolkata. There is hardly any demarcation of areas of distinct residential, industrial, commercial activities. The city area under the Kolkata Municipal Corporation covers an area of 187 km^2^ of which only 6–7 % of land is used for road space. Vehicular density of the city is 5685 cars/km^2^ and average traffic speeds is less than 20 km/h. Number of registered vehicles in the city is projected to about 1.3 million by 2015 [19, 20]. An area within the four important traffic intersections of south Kolkata i.e., Park Street (22°33′17.23″N, 88°21′50.14″E), Park Circus (22°32′35.82″N, 88°21′58.14″E), Garia (22°27′57.08″N, 88°22′40.10″E) and Tollyguange Tram Depot (22°29′35.10″N, 88°20′43.04″E) was chosen for road-traffic characteristics and noise survey.

### Comprehensive study plan for data collection

**Fig. 1:**
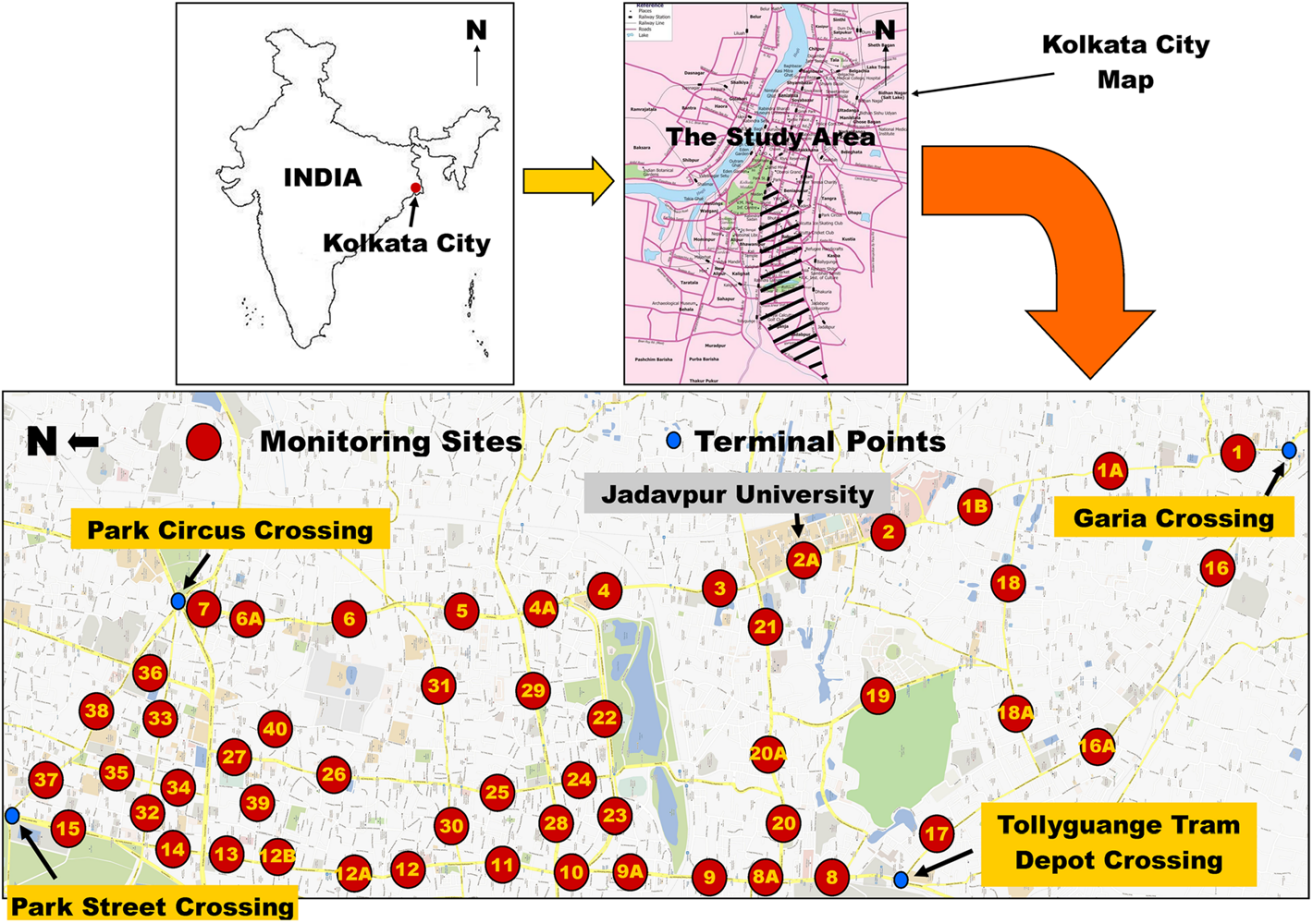
Study area and locations for traffic volume and noise survey

Total 280 sets of data were generated on the following variables during two phases of monitoring programme. In the first phase, motorized traffic volume and traffic noise level were monitored for 4 h at curbside open-air microenvironment of 23 major roads of the study area. In this phase traffic volume and noise monitoring were performed once between 12:00 noon and 04:00 p.m. at 52 sites on different week days of March’2011–May’2011. In the second phase, traffic volume and noise monitoring were performed at 38 sites located on 21 major roads of the study area. In this phase no monitoring was performed at the site number 39 and 40 as well as A and B sub-sites of the respective site number (Fig. 1). At each monitoring site three consecutive 4 h monitoring of traffic volume and noise were performed on two different consecutive week days. On the first day the monitoring were performed between 04:00 p.m.–08:00 p.m. and on the second day the monitoring were performed between 08:00 a.m.–12:00 noon and 12:00 noon–04:00 p.m. Three consecutive 4 h monitoring at each site were also repeated in summer of the year 2012 (March’2012–May’2012) and 2013 (March’2013–May’2013) and winter of the year 2011–2012 (November’2011–February’2012) and 2012–2013 (November’2012–February’2013). In both the phases data on meteorology corresponding to each set of traffic volume and noise monitoring was recorded from a roof top automated weather monitoring station with in the study area. Road width of the monitoring sites was estimated once with a measuring tape. No monitoring was performed in monsoon and rainy day.

### Monitoring of traffic noise level

Traffic noise level of the microenvironment was determined in terms of 4 h A-weighted equivalent noise level (L_eq_) with a Type-II (SC160, CESVA make) sound level meter (SLM). The SLM was operated under fast operation mode with 1 s resolution. It was placed on a tripod, on road side walk, at a distance of 1 m from boundary wall and at a height of 1.5 m from ground level. It was also calibrated prior to each 4 h monitoring. Noise monitoring was strictly avoided near constructional activities. Statistical noise levels or n-percent exceeded noise levels (L_n_, where *n* = 1, 5, 10, 50, 90, 95 and 99) were also determined from the SLM and grouped as peak (L_1_, L_5_, L_10_), background (L_90_, L_95_, L_99_) and median (L_50_) noise level for statistical analysis [21, 22].

### Monitoring of motorized traffic volume

Motorized traffic volume was determined on analysis of 15 min video footage taken once in an hour during total 4 h noise monitoring with a digital camera (DSC-W150, Sony make). Traffic volume was determined on manual counting of vehicles passed through the cross section of the road observed through the digital camera. Then hourly traffic volume was determined with a multiplication factor of 4. Finally the motorized traffic volume was represented as vehicles/4 h by simple addition of each 1 h data.

### Monitoring of meteorology

Four hour averaged data on meteorological variables like, air temperature, relative humidity and wind speed corresponding to each set of the traffic volume and noise monitoring were recorded from a roof-top automated weather monitoring station (WM 250, Envirotech make) placed at the Jadavpur University campus.

### Computation of noise indices

Noise indices like, Noise Climate (NC) and Traffic Noise Index (TNI) were computed according to the following equations:

1 $$ NC={L}_{10}-{L}_{90} $$

2 $$ TNI=\left({L}_{90}+4\times NC-30\right) $$

Where, L_10_ and L_90_ is the traffic noise level exceeded for 10 and 90 % of the sampling time respectively.

### Statistical analysis

Central tendency of the variables was determined through descriptive statistical parameters like, minimum, maximum, range (difference between the maximum and the minimum), mean, standard deviation (SD) and coefficient of variation (CV). Descriptive statistical analysis was performed in Statistical Package for the Social Sciences (SPSS) v20 environment. CV was determined with the following equation:

3 $$ CV=\frac{\sigma_X}{\mu_X}\times 100 $$

Where, σ_x_ is the standard deviation of the variable X and μ_x_ is the mean of the variable X. CV was used as a tool to measure the level of spatio-temporal heterogeneity of a variable. Pearson’s correlation coefficients were calculated between meteorological variable(s) and traffic noise level to determine the effect of meteorology on the prevailing traffic noise level at the microenvironment. A Varimax rotated principal component analysis (PCA) with Kaiser normalization was also performed to explore the relationship of road width, motorized traffic volume, traffic noise level and peak, median and background noise level at the microenvironment. The PCA was also performed in SPSS v20 environment according to the manual “Solving Homework Problems in Data Analysis II” [23].

## Results and discussion

### Heterogeneity of the environmental condition

Range of motorized traffic volume was accounted for 16,592 vehicles/4 h with a mean of 9619 ± 3367 vehicles/4 h. Spatio-temporal heterogeneity of traffic volume was accounted for 35 %. Range of road width was accounted for 24 m with a mean of 18.4 ± 6.4 m. Spatial heterogeneity of road width were comparable with the same of traffic volume (Table 1). This might be attributed to a higher traffic volume in wider roads.

**Table 1:** Environmental condition during monitoring

Variables	Minimum	Maximum	Mean	SD	CV, %
Vehicles/4 h	3228	19820	9619	3367	35
Road width, m	7.0	31.0	18.4	6.4	34.8
Air temperature, °C	12.8	39.3	28.6	6.3	21.9
Relative humidity, %	20.3	89.3	57.3	13.0	22.7
Wind speed, km/h	0.0	28.7	11.1	5.9	53.0

Range of air temperature was accounted for 26.5 °C with a mean of 28.6 ± 6.3 °C during the study period. Temporal heterogeneity of air temperature was of 21.9 %. Range of relative humidity was accounted for 69 % with a mean of 57.3 ± 13 %. Observed temporal heterogeneity of relative humidity was of 22.7 % which was similar to air temperature. But wind speed had the highest degree of temporal heterogeneity of 53 % among the meteorological variables. Range of wind speed was accounted for 28.7 km/h with a mean of 11.1 ± 5.9 km/h.

### Traffic noise level and annoyance response

Range of traffic noise level in the microenvironment was accounted for 13.1 dB(A) with a mean of 77.6 ± 2.1 dB(A). Spatio-temporal heterogeneity of traffic noise level was of 2.7 % which indicated homogeneous spatio-temporal distribution of traffic noise level in spite of marked spatio-temporal heterogeneity of motorized traffic volume at the monitoring locations (Table 2). Chowdhury et al. also reported a similar finding in the context of Kolkata city, India [24]. It was also noteworthy that the recorded minimum traffic noise level was well above the prescribed standards of 65 dB(A) for day time for commercial area recommended by the Central Pollution Control Board (CPCB) of India [25]. Minimum and maximum exceedance of traffic noise level from the standard were accounted for 5.3 dB(A) and 18.3 dB(A) respectively. The average exceedance of prevailing traffic noise level at the microenvironment from the standard was of 12.6 ± 2.1 dB(A). Very high noise level at the microenvironment in the context of different Indian cities has been reported by several researchers [13–16]. Peak (L_1_, L_5_ and L_10_) and median (L_50_) noise levels had almost equal and lower degree of spatio-temporal heterogeneity but background (L_90_, L_95_ and L_99_) noise levels had higher degree of spatio-temporal heterogeneity at the microenvironment of Kolkata city.

**Table 2:** Traffic noise level and annoyance response to traffic noise

Variables	Minimum	Maximum	Mean	SD	CV, %
L_eq_, dB(A)	70.3	83.4	77.6	2.1	2.7
L_1_, dB(A)	81.0	95.7	88.5	2.5	2.9
L_5_, dB(A)	75.4	88.2	82.5	2.2	2.7
L_10_, dB(A)	72.8	85.0	79.8	2.0	2.5
L_50_, dB(A)	66.0	77.1	72.7	2.1	2.8
L_90_, dB(A)	60.6	72.0	66.8	2.3	3.5
L_95_, dB(A)	59.0	70.6	65.3	2.4	3.7
L_99_, dB(A)	52.3	69.0	62.9	2.6	4.1
NC, dB(A)	8.8	18.8	13.0	1.8	13.7
TNI, dB(A)	75.3	107.4	88.8	6.1	6.9

Annoyance response to traffic noise level was estimated in terms of NC and TNI. NC represents the difference between peak and background noise level. Higher values of peak noise level and lower values of background noise level resulted higher values of NC. Higher numerical values of NC represent an annoying environment. Range of NC was accounted for 10 dB(A) with a mean of 13 ± 1.8 dB(A). Better and comparable NC in the context of Indian cities has been reported from Kolhapur City and Chidambaram Town [14, 26]. Worse NC in the context of Indian cities has been reported from Baripada Town and Rourkela City [27, 28]. TNI over 74 dB(A) is defined as threshold of over criterion and was found sufficient to create annoyance among people [29]. It was noteworthy that the minimum TNI was accounted for 75.3 dB(A). This implied annoyance response to traffic noise level at the microenvironment of Kolkata City was very high. TNI over 74 dB(A) have also been reported from the Indian towns and cities like Chidambaram, Baripada, Rourkela and Gwalior [26–28, 30]. Spatio-temporal heterogeneity of NC was found higher than TNI.

### Relationship of meteorology and traffic noise level

**Fig. 2:**
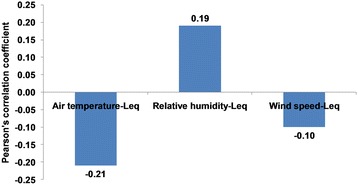
Relationship of meteorology and equivalent noise level

A weak negative correlation of −0.21 (*p* < 0.01) was accounted between air temperature and prevailing traffic noise level at the microenvironment (Fig. 2). The correlation might be attributed to higher pavement temperature which co-occurs with higher air temperature [31]. Reduction in traffic noise level to proximity of a road due to higher pavement temperature was also accounted in the research work of Anfosso-LédéE et al. and Bueno et al. [32, 33]. On the contrary a very weak positive correlation of 0.19 (*p* < 0.01) was accounted between relative humidity and prevailing traffic noise level at the microenvironment. Positive relationship between the two variables has also been reported in the research work of Subramani et al. and Pachiappan et al. in the context of Coimbatore and Salem City [34, 35]. Wind speed had very weak negative correlation of −0.10 (statistically not significant) with equivalent traffic noise level at the microenvironment. Negative correlation between traffic noise level and wind speed might be attributed to higher atmospheric turbulence during high wind hours. Higher atmospheric turbulence may scatter sound unpredictably which results a reduction in sound level close to road [36]. It is clear from the above discussion that meteorology had little influence on prevailing traffic noise level at the microenvironment of the city. Insignificant influence of meteorology on traffic noise level close to road was also reported in the context of Essen [37]. For this reason the meteorological variables were exempted in principal component analysis.

### Relationship of traffic volume, road width, peak, median and background noise and equivalent noise level

**Fig. 3:**
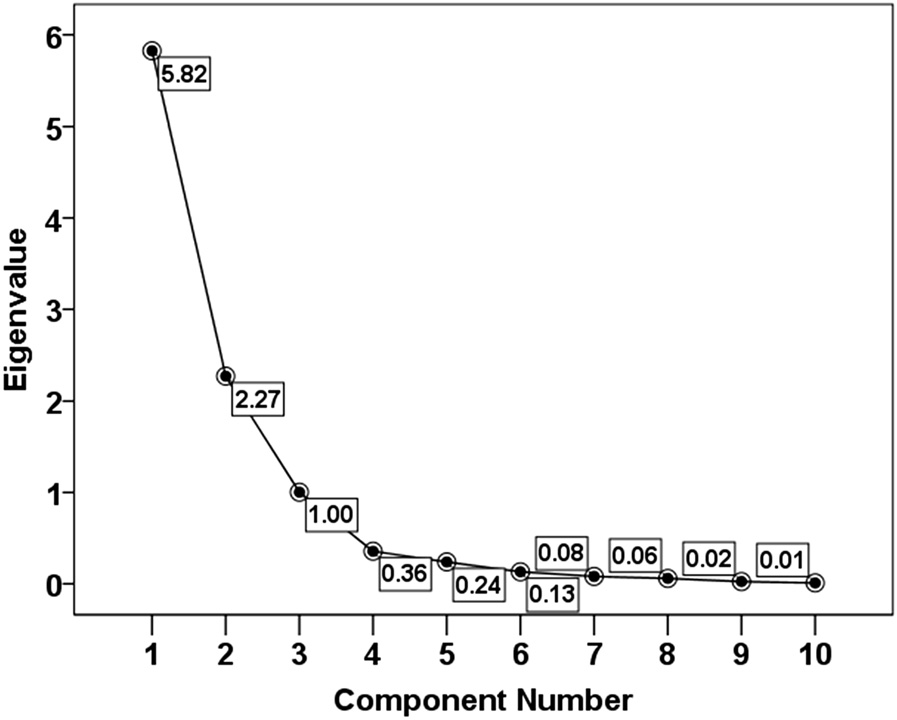
Scree plot of the extracted components

Three components of Eigenvalue >1 were extracted through the principal component analysis (Fig. 3; Table 3). The Component-1, Component-2 and Component-3 explained respectively 58.25, 22.73 and 10.03 % of the variance in the data set.

**Table 3:** Validation of principal component analysis

Control points recommended for validation of a principal component analysis [23]	Observed
The sample size must be greater than 50	280
The ratio of cases to variables must be 5 to 1 or larger	28 to 1
The correlation matrix for the variables must contain 2 or more correlations of 0.30 or greater	31
Variables with measures of sampling adequacy less than 0.50 must be removed	Nil
The overall measure of sampling adequacy is 0.50 or higher	0.80
The Bartlett test of sphericity is statistically significant.	Significant at the *p* < 0.001 level
The derived components explain 50 % or more of the variance	91.01 %
Communality of the individual variables less than 0.50 should be removed	Nil
None of the components has only one variable in it	Nil

L_90_, L_95_ and L_99_ i.e., background noise levels had very strong positive loading (>0.80) with The Component-1 (Table 4). Therefore, the component may be termed as background noise component. Motorized traffic volume had moderate positive loading of with this component. Therefore, the background noise level might be attributed to consistent and relatively lower level of noise which originates from road-tyre interactions and engine of the moving vehicles.

**Table 4:** Loading of the variables with the components

Variables	Component-1	Component-2	Component-3
Vehicles/4 h	0.44	−0.01	0.82
Road width, m	−0.01	−0.12	0.94
L_eq_, dB(A)	0.31	0.91	−0.08
L_1_, dB(A)	0.05	0.88	−0.17
L_5_, dB(A)	0.30	0.93	−0.02
L_10_, dB(A)	0.43	0.87	0.10
L_50_, dB(A)	0.75	0.52	0.19
L_90_, dB(A)	0.92	0.32	0.10
L_95_, dB(A)	0.95	0.24	0.11
L_99_, dB(A)	0.92	0.17	0.12

L_1_, L_5_, L_10_ i.e., peak noise levels had very strong positive loading with the Component-2. Therefore, the component may be termed as peak noise component. Significantly traffic noise level (L_eq_) had very strong positive loading with this component. Therefore prevailing traffic noise level is directly proportionate to the peak noise level at the microenvironment of the city. The peak noise level is manageable to some extent because it is almost behavioural and originates mainly due to honking, sudden acceleration and deceleration of vehicles. Vijay et al. reported that no honking may reduce the traffic noise level by 2 to 5 dB(A) at the microenvironment [18]. Significantly L_50_, the median noise level had ambiguous relationship with both background and peak noise component but the degree of linearity was higher with the background noise component. This might be attributed to median noise level is free from the influences of instantaneous short lived peak noise level [38].

Motorized traffic volume and road width had very strong positive loading with Component-3. This might be attributed to higher traffic volume in wider roads. Probably due to this reason equally higher degree of spatial heterogeneity was observed for road width and motorized traffic volume.

## Conclusions

Noise is not uniformly distributed in different urban settings. Curbside open-air microenvironment of a city is one of the predictable settings with excess noise level and it disproportionately impacts individuals living in these areas. Traffic noise level at curbside open-air microenvironment of Kolkata city was well above the standard prescribed by CPCB of India. It was also highly annoying. Air temperature and relative humidity had little influence and the peak noise component had the most significant influence on the prevailing traffic noise level of the microenvironment. Peak noise level is manageable to some extent because it is almost behavioural and originates mainly due to honking, sudden acceleration and deceleration of vehicles. Therefore, traffic noise level of Kolkata city at curbside open-air microenvironment can be reduced with careful honking and driving.

## Abbreviations

CPCB: Central Pollution Control Board
L_eq_: Equivalent noise level
SLM: Sound level meter
L_n_: n-percent exceeded noise levels
NC: Noise Climate
TNI: Traffic Noise Index
SD: Standard deviation
CV: Coefficient of variation
SPSS: Statistical Package for the Social Sciences
PCA: Principal component analysis
